# Necrotic Primary Central Nervous System Lymphoma in an Immunocompetent Patient: A Case Report and Literature Review

**DOI:** 10.7759/cureus.4910

**Published:** 2019-06-17

**Authors:** Peter J Fiester, Erik Soule, Patrick E Natter, Dalys Haymes, Dinesh Rao

**Affiliations:** 1 Neuroradiology, University of Florida Health, Jacksonville, USA; 2 Interventional Radiology, University of Florida College of Medicine, Jacksonville, USA

**Keywords:** immunocompetent, magnetic resonance imaging, primary central nervous system lymphoma, diffusion weighted imaging, computed tomography

## Abstract

Primary lymphoma that arises de novo from the central nervous system (CNS) is most commonly a non-Hodgkin's B-cell lymphoma and by definition lacks the presence of disease outside the CNS. It demonstrates a characteristic imaging appearance on computed tomography (CT) and magnetic resonance imaging (MRI) exams related to its inherent hypercellularity. On CT, primary CNS lymphoma (PCNSL) demonstrates a hyperdense appearance; on MRI, it commonly demonstrates restricted water diffusion on diffusion-weighted sequences and homogeneous enhancement on post-contrast sequences. We present a case of primary CNS lymphoma in an immunocompetent patient with progressive necrosis and loss of restricted diffusion on diffusion-weighted imaging (DWI) with an atypical enhancement pattern. We further provide a review of the literature regarding the CT and MRI appearance of primary CNS lymphoma and discuss the role of immune status in determining the imaging characteristics of this disease process.

## Introduction

Lymphoma may occur as a de novo primary central nervous system (CNS) lymphoma (PCNSL) with absence of disease outside the CNS or as a secondary CNS lymphoma (SCNSL) related to systemic disease and CNS metastasis [1-2]. Both PCNSL and SCNSL are most commonly of B cell origin with 90%-95% of PCNSL and 80% of SCNSL presenting as a B-cell non-Hodgkin's lymphoma [1-3]. The precise origin of PCNSL is debated since the CNS lacks a lymphatic system and lymphocytes; however, PCNSL does demonstrate increased incidence in immunocompromised patients with human immunodeficiency virus (HIV) or acquired immunodeficiency syndrome (AIDS). This is postulated to be related to the activation of the Epstein-Barr virus (EBV) [1,3].

Whereas SCNSL typically involves the skull and dura, PCNSL much more commonly involves the brain parenchyma and has a characteristic imaging appearance in the immunocompetent patient population related to the tumor’s inherent hypercellularity, high nuclear/cytoplasmic ratio, and disruption of the blood-brain barrier. On non-contrast computed tomography (CT), PCNSL is typically hyperdense. When magnetic resonance imaging (MRI) is performed, PCNSL demonstrates diffusion restriction on diffusion-weighted imaging (DWI) and avid enhancement with gadolinium administration. PCNSL has a predilection for the deep structures of the brain and typically demonstrates a periventricular or perivascular location. Two-thirds of tumors present as a solitary mass. The presence of necrosis and hemorrhage with PCNSL is uncommon unless the patient is immunocompromised [1,4-5].

## Case presentation

A 50-year-old male with a past medical history of hypertension and heavy alcohol use in the past (reportedly abstinent for the preceding four months) presented with one month of recurrent and progressive dizziness and worsening memory impairment. The patient also endorsed anomic aphasia (word finding difficulty), acalculia (difficulty with simple calculations), and confusion. Neurologic exam demonstrated 3+ hyperactive lower extremity reflexes, a positive Babinski sign bilaterally, decreased lower extremity vibratory sensation, and conjugate nystagmus on lateral gaze with horizontal diplopia. The remaining neurologic exam was unremarkable. 

Based on presenting signs and symptoms, the patient was tentatively diagnosed with Wernicke-Korsakoff syndrome. Laboratory testing, including comprehensive metabolic panel, complete blood count, thyroid stimulating hormone, HIV, and thiamine level were performed. Laboratory findings revealed an elevated white blood cell count of 12.6 × 10^9^/L and a normal thiamine level; remaining laboratory findings were within normal limits. A lumbar puncture with cerebrospinal fluid (CSF) analysis was unremarkable. Non-contrast head CT followed by serial MRI examinations of the brain without and with intravenous (IV) gadolinium was performed for further assessment.

**Figure 1:**
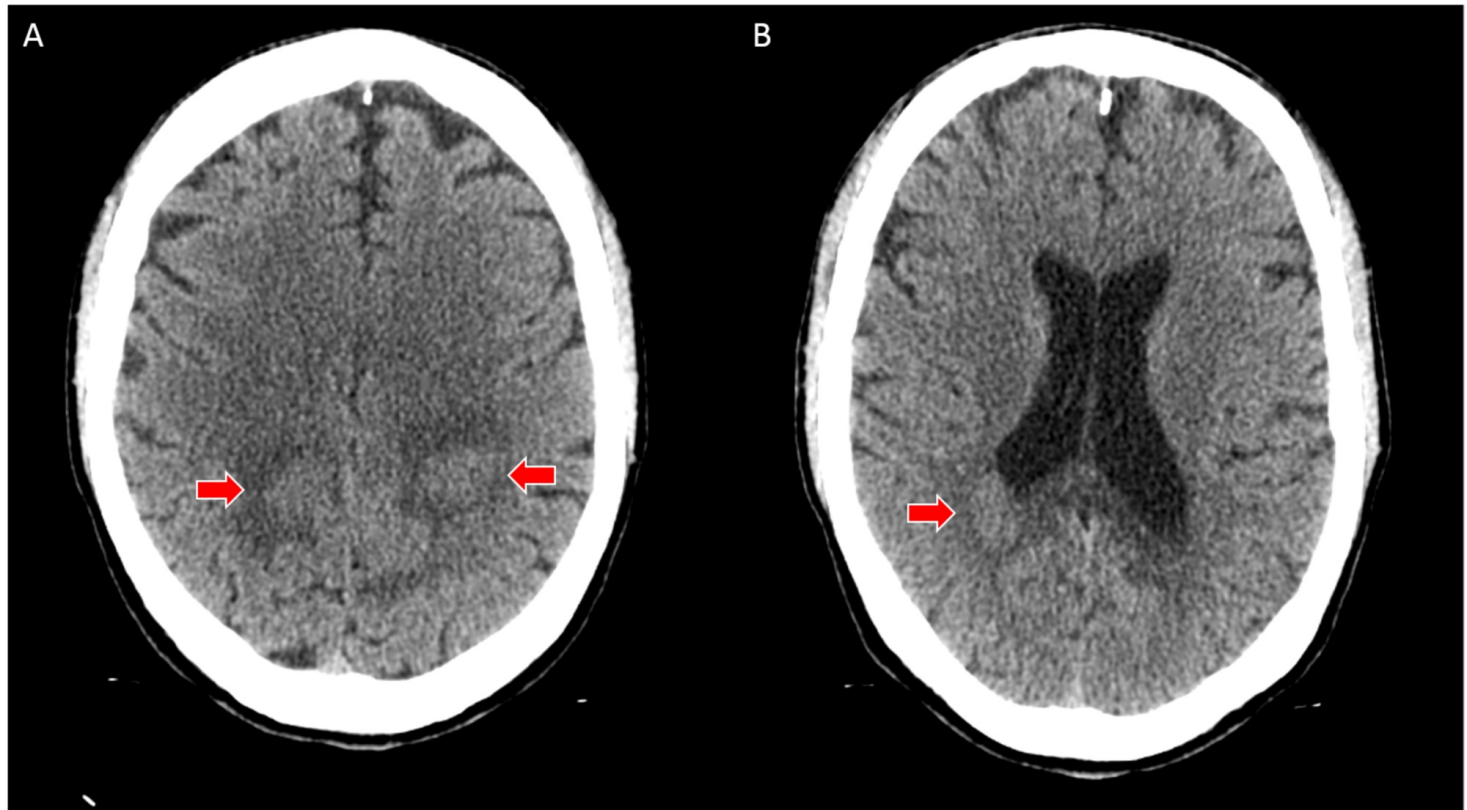
A - axial computed tomography (CT) image demonstrates a slightly hyperdense mass with adjacent edema involving the parietal white matter (red arrows). B - axial CT image demonstrates a slightly hyperdense mass with adjacent edema involving the splenium of the corpus callosum (red arrow)

Initial non-contrast CT of the head demonstrated a well-demarcated area of slightly hyperdense attenuation with surrounding edema centered in the bilateral parietal lobes and splenium of the corpus callosum extending across the midline. There was no hemorrhage or mass effect (Figure 1).

**Figure 2:**
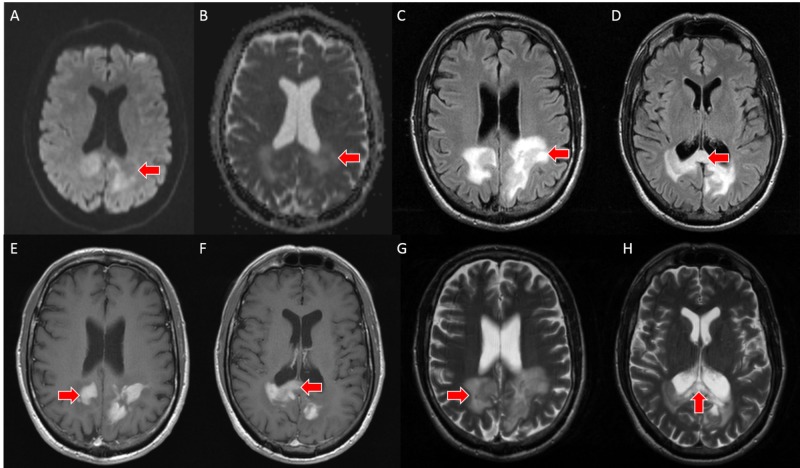
A - brain MRI with DWI sequence demonstrates mild restricted water diffusion associated with a mass in the bilateral parietal lobes and splenium (red arrow). B - brain MRI with ADC mapping demonstrates mild restricted water diffusion associated with a mass in the bilateral parietal lobes and splenium (red arrow). C, D - axial slices from brain MRI with FLAIR sequence demonstrates perilesional vasogenic edema within the splenium of the corpus callosum and in the adjacent parietal lobes (red arrows). E, F - axial slices from brain MRI with T1-weighted post-contrast sequence demonstrate discontinuous, homogeneous enhancement (red arrows). G, H - axial slices from brain MRI with T2 sequence demonstrate an isointense mass in the splenium of the corpus callosum and in the adjacent parietal lobes with surrounding vasogenic edema (red arrows) MRI: magnetic resonance imaging, DWI: diffusion-weighted imaging, ADC: apparent diffusion coefficient, FLAIR: fluid-attenuated inversion recovery.

Based on the head CT findings and clinical presentation, a follow-up pre and post-contrast brain MRI was performed and demonstrated a mass in the subcortical white matter in the bilateral parietal lobes and splenium of the corpus callosum with extension across the midline and surrounding vasogenic edema. The mass was isointense on T2-weighted and fluid-attenuated inversion recovery (FLAIR) sequences. DWI with a B-value of 1000 and corresponding apparent diffusion coefficient (ADC) map demonstrated mild diffusion restriction. The post-contrast T1-weighted sequence demonstrated discontinuous areas of homogeneous enhancement. No associated mass effect, volume loss, or hemorrhage was present (Figure 2).

**Figure 3:**
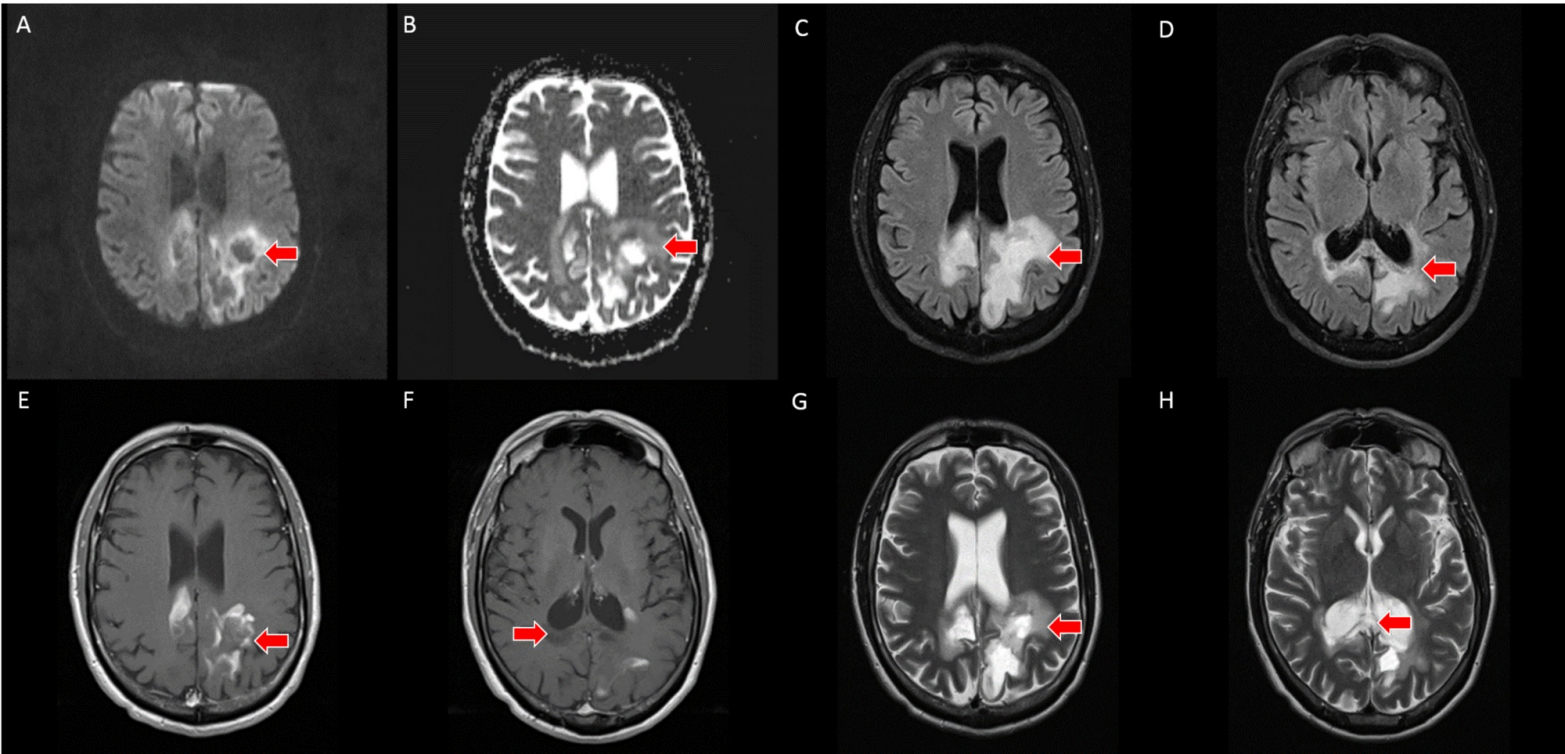
A, B - axial slices from three-month follow up brain MRI with provided DWI/ADC sequences (top left) demonstrate T2 shine through without restricted water diffusion (red arrows) in the region of previously seen restricted water diffusion. C, D - axial slices from three-month follow up brain MRI with FLAIR sequences demonstrate necrosis and cystic change within the corpus callosum and subcortical white matter of the bilateral parietal lobes (red arrows). E, F - axial slices from three-month follow up T1-weighted post-contrast brain MRI demonstrate resolution of the homogeneous enhancement with an incomplete, peripheral enhancement pattern. G, H - axial slices from three-month follow up brain MRI with T2-weighting demonstrate necrosis and cystic change within the corpus callosum and subcortical white matter of the bilateral parietal lobes (red arrows) MRI: magnetic resonance imaging, DWI: diffusion-weighted imaging, ADC: apparent diffusion coefficient, FLAIR: fluid-attenuated inversion recovery.

**Figure 4:**
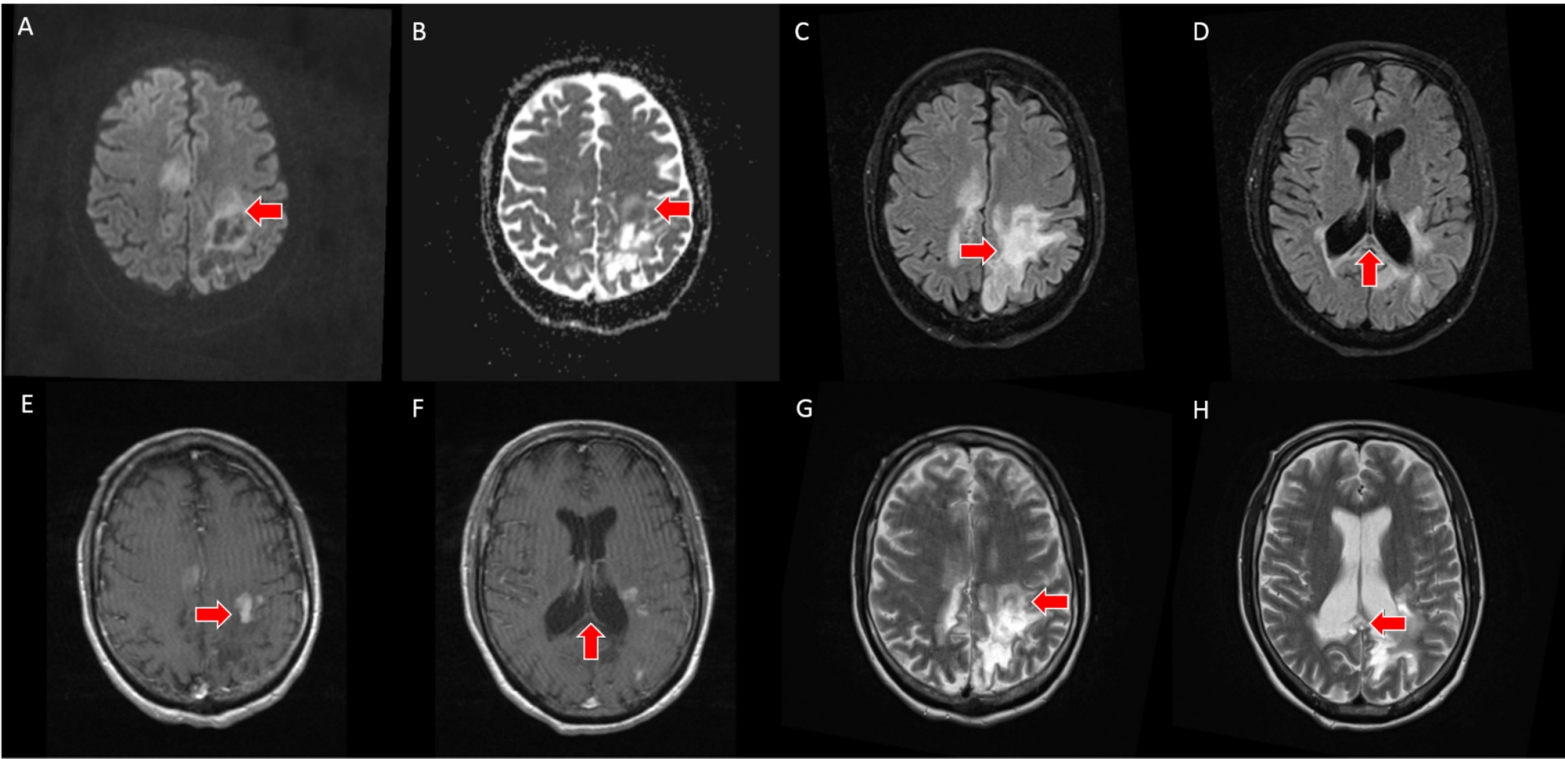
A, B - axial slices from five-month follow-up brain MRI demonstrates lack of restricted diffusion on DWI/ADC. C, D - axial slices from five-month follow-up brain MRI with FLAIR sequence demonstrate increased necrosis and cystic change. E, F - axial slices from five-month follow-up contrast enhanced T1-weighted brain MRI demonstrate a scattered, leading edge enhancement pattern. G, H - axial slices from five-month follow-up T2-weighted brain MRI demonstrate increased necrosis and cystic change MRI: magnetic resonance imaging, DWI: diffusion-weighted imaging, ADC: apparent diffusion coefficient, FLAIR: fluid-attenuated inversion recovery.

PCNSL was suspected based on the imaging appearance; however, the patient declined biopsy and further treatment at that time. The patient slowly developed worsening neurologic symptoms, and subsequent follow-up pre- and post-contrast brain MRI exam was performed at three and five-month intervals. T2-weighted imaging demonstrated progressive necrosis and cystic change within the corpus callosum and bilateral parietal white matter. DWI demonstrated T2 shine through in the areas of previously seen restricted water diffusion, and T1-weighted post-contrast imaging demonstrated a peripheral incomplete ring of enhancement (Figures 3-4).

**Figure 5:**
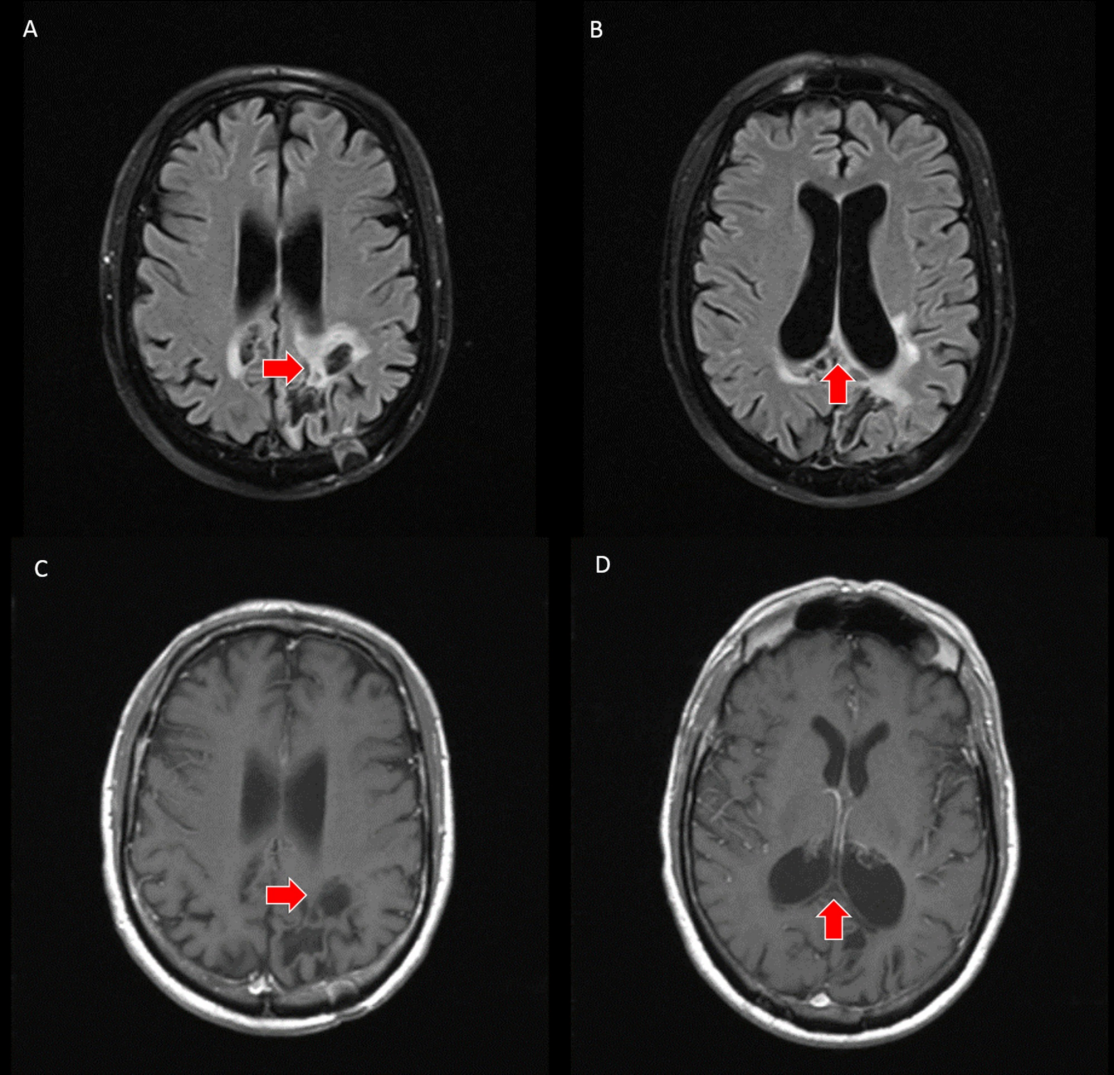
A, B - axial slices from brain MRI following chemotherapy with FLAIR-weighted sequence demonstrates decreased volume of the lesion (red arrows). C, D - axial slices from brain MRI following chemotherapy with T1-weighted pre and post-contrast sequences demonstrate resolution of the previously seen enhancement (red arrows) MRI: magnetic resonance imaging, FLAIR: fluid-attenuated inversion recovery.

The question of whether this lesion represented tumefactive demyelination (TDL) was raised based on the follow-up imaging. The patient subsequently underwent a left occipital-approach stereotactic biopsy of the lesion. The specimen yielded sheets of large lymphocytes with open chromatin, nucleoli and high nuclear to cytoplasmic ratio consistent with diffuse large B-cell lymphoma. Inpatient treatment with chemotherapy was initiated. Post treatment brain MRI demonstrated a positive response to therapy with decreased volume of the lesion and resolution of the peripheral enhancement (Figure 5).

## Discussion

This case is unique for several reasons. Serial follow-up brain MRI exams demonstrate intercurrent lack of hypercellularity associated with the tumor as no restricted water diffusion on DWI was present at the three and five month intervals. A study by Haldorsen et al.reviewed the CT and MRI findings of 76 patients with biopsy-proven PCNSL and found 100% of their patients demonstrated either isodense or hyperdense attenuation of the tumor relative to white matter on non-contrast head CT [6]. Furthermore, Mansour et al.reviewed the MRI appearance of PCNSL in 21 immunocompetent patients and found either restricted water diffusion or isointense signal within the tumor on DWI [7]. However, this imaging appearance on CT and MRI exams may be more common in low-grade PCNSL (defined by the predominance of small, mature lymphocytes and a growth fraction < 20%). In fact, Schobet al. demonstrated a significant correlation between low ADC levels and higher proliferative activity of PCNSL [8].

The serial follow-up MRI exams performed in this patient who refused biopsy and surgical treatment also demonstrated an increasingly necrotic appearance of the tumor on T2-weighted imaging with internal areas of hyperintense signal intensity within the corpus callosum and biparietal deep white matter. This imaging appearance on MRI has been observed in AIDS-related PCNSL with many of the lesions exhibiting necrotic regions and more commonly a multifocal or disseminated appearance [1,5,9]. Subsequent MRI exams also demonstrated a peripheral “leading edge” of enhancement. Intrinsic enhancement within PCNSL results from disruption of the blood brain barrier and accumulation of contrast material within the tumor. PCNSL in immunocompetent patients has a characteristic avid and homogeneous enhancement pattern. In all 21 patients with PCNSL, Mansour et al. describe positive enhancement pattern and most commonly avid enhancement [4]. Haldorsen et al.describe homogeneous contrast enhancement on MRI in 90% of patients in the non-AIDS population [5]. 

Atypical MRI appearance of PCNSL (eg necrosis, irregular or peripheral enhancement) in an immunocompetent patient is rare in the USA. This presentation is more common among patient with tumors who are positive for EBV [9-10]. EBV-positive PCNSL is more common in males and patients > 60 years old who are immunocompromised due to conditions such as HIV, chronic alcoholism, and several collagen vascular diseases. Recent studies, however, have shown that this process may be also be related to immunologic deterioration associated with the normal aging process [9,10]. Several studies have postulated a relationship between EBV-positive PCNSL and disease survival. One study concluded that patient with EBV-positive PCNSL had shorter overall survival than EBV-negative PCNSL [11].

Given the imaging appearance on follow-up brain MRI exams, the question of whether this lesion could represent tumefactive demyelination (TDL) was also raised. Differentiating PCNSL from TDL may be extremely difficult in some patients due to the variability of clinical presentation and imaging findings [12]. The lymphodepletive effect of corticosteroid drugs, which is routinely given to patients with suspected TDLs before the biopsy, can obscure the histologic features of both PCNSLs and TDLs [13]. Corticosteroids can also further confound the diagnostic dilemma by obscuring radiologic findings of both TDLs and PCNSLs [12-13]. Demyelination may also be associated with a paraneoplastic phenomenon, since anti-myelin oligodendrocyte glycoprotein (anti-MOG) antibodies have been found in the serum of patients with sentinel demyelinating lesions preceding the presentation of PCNS lymphoma [11].

Histologically, differentiating demyelination from PCNSL may also be challenging due to the fact the PCNSL may present in association with steroid-responsive multifocal demyelinating sentinel lesions, which histologically are indistinguishable from multiple sclerosis (MS), containing predominantly T-cell infiltrates and a few B cells [8,11]. Additionally, abnormal mitotic figures in reactive astrocytes within TDLs can potentially mimic high-grade gliomas on histology. One author postulates that T-cell infiltrates represent a cell-mediated anti-tumor response to B-cell lymphoma [11,14-15]. This observation is derived from reports of spontaneous regression of lymphomas in immunocompetent individuals as well as improved survival in follicular lymphoma [16].

A few studies have tried to differentiate TDLs from PCNSLs using advanced MR imaging techniques. Two studies demonstrated a significantly higher ADC minimum in TDL than in PCNSL with one study demonstrating that ADC minimum with a threshold of 556 × 10^−6 ^mm^2^/s was the best indicator for differentiating TDL from atypical PCNSL (a sensitivity of 81% and specificity of 89%) [17-18].

Another study showed a lower choline/N-acetylaspartate (NAA) ratio in TDLs than in PCNSLs, with a threshold for differentiation of 1.73 yielding sensitivity of 89% and specificity of 76%. In addition, non-contrast CT hypoattenuation of MRI enhancing regions was observed in 93% of TDL cases, but only 4% of primary brain tumors [19]. One study revealed that the combination of conventional MR imaging and advanced MR imaging improved the diagnostic performance for differentiating TDL from PCNSLs or high-grade gliomas [19]. ADC values, MR spectroscopy, and non-contrast CT may help in diagnosing TDLs; however, further study is required to determine the added value of advanced MR imaging techniques in the differentiation of TDLs from PCNSLs [9]. Susceptibility-weighted imaging (SWI) may provide additional information when evaluating patients with atypical PCNSL. Lee et al. demonstrated a considerable frequency of hemorrhage (18%) in immunocompetent patients with PCNSL, with significant predominance among patients with EBV-positive PCNSL vs. EBV-negative PCNSL (70% vs. 7%) [12].

## Conclusions

We present an unusual case of PCNSL in an immunocompetent patient demonstrating a progressive, atypical imaging appearance on MRI. Findings help reinforce the protean appearance of PCNSL, which may occasionally lack the characteristic hypercellular imaging appearance on CT and MRI exams especially if left untreated. Review of the literature suggests the presence of necrosis and peripheral enhancement on MRI to be uncommon in the immunocompetent population and more commonly associated with EBV-positive tumors or in HIV positive patients. The lack of the typical imaging appearance of PCNSL should not dissuade one from the diagnosis, especially for a lesion located in the deep white matter or corpus callosum.
